# Utilization and performance of long-term care system for older people with disabilities and dementia in Zhejiang Province, China

**DOI:** 10.3389/fpsyt.2023.1148682

**Published:** 2023-03-24

**Authors:** Tongda Sun, Xiuli Liu, Wenyin Jiang, Xiaoxin Dong, Minmin Jiang, Lu Li

**Affiliations:** ^1^Institute of Health Service Research, Ningbo College of Health Sciences, Ningbo, China; ^2^Shulan International Medical College, Zhejiang Shuren University, Hangzhou, China; ^3^Center for Modern Service Industry Research of Zhejiang Province, Hangzhou, China

**Keywords:** older people with disabilities and dementia, long-term care system, utilization, performance, evaluation indicator system, public health, public policy

## Abstract

**Introduction:**

To explore changes in performance, weaknesses, and utilization of the long-term care (LTC) system for older people with disabilities and dementia (OPWDD) in Zhejiang Province, China, thereby providing a reference for decision-making amid a progressively aging population.

**Methods:**

A performance evaluation model of the LTC system for OPWDD was constructed using three dimensions: input, process, and outcome. Performance indicators and trends were calculated based on data collected from statistical yearbooks, documents, and work reports of the Bureau of Statistics and other government departments in Zhejiang Province, China, published in 2015–2021.

**Results:**

Significant improvements were observed in most LTC performance indicators for OPWDD, such as input, process, and outcome, with notable enhancements in fairness, accessibility, and affordability of LTC services. By 2021, there were 6.20 nursing and rehabilitation beds in medical institutions and 3.77 general practitioners per 1,000 people aged 65 and above, up 144.14% and 13.73%, respectively, from 2015. The rate of health management for older people was 70.91%, representing a 10.33% increase from 2015. The actual reimbursement ratio of hospitalization expenses covered by basic medical insurance for older people rose 7.05%, from 72.76% in 2015 to 77.89% in 2021. Social security satisfaction rose 12.4%, from 71.3% in 2015 to 83.7% in 2021. Certain indicators, however, showed no significant improvement and tended to decline, such as the number of beds at older care institutions and caregivers per 1,000 people aged 65 and over.

**Discussion:**

It is imperative to further balance the allocation of care resources, using a people-centered and integrated LTC system. The proportion of rehabilitation and nursing beds for older people should be consistently increased to effectively alleviate the shortage of care beds. Furthermore, a talent incentive policy should be improved to train caregivers and provide whole-person and whole-life course care based on OPWDD needs.

## 1. Introduction

Since the end of the 20th century, the proportion of older people and their percentage in the overall population has been consistently growing in China. The number of people aged 60 and over in China rose from 126 million in 2000 to 267 million in 2021, with the proportion of older people in the total population growing from 10.2 to 18.9%. It is estimated that in the 14th 5-Year Plan period (2021–2025), the number of people aged 60 and over in China is expected to exceed 300 million, accounting for more than 20% of the population. By 2035, it is expected that there would be about 420 million people aged 60 and over in China, constituting more than 30% of total population. The number of people aged 65 and over is expected to grow from 200.56 million in 2021 to 366 million in 2050, representing an increase from 14.2 to 26.1% of the total population (1–4). The percentage of people aged 65 and over in the total population is expected to continue to increase in the coming decades, more than doubling from 11.5% in 2019 to 26.1% in 2050, in China. Compared to Organisation for Economic Cooperation and Development (OECD) member-countries, China’s rate of increase is higher, rising from 17.3% in 2019 to 26.7% by 2050 (5). China is also expected to witness a rapid increase in the number of OPWDD during this period. China reported 52.71 million older people with disabilities in 2020, and the figure is expected to increase to 136.74 million by 2030 (6). In 2015, China ranked first in the world with about 9.5 million people living with dementia (PLWD), accounting for about 25% of global cases of dementia among older people. This figure is expected to double to 16.2 million by 2030 (7, 8).

Disabilities and dementia are among the primary reasons of care dependency among older people. Considering that caregiving places a heavy burden on families of OPWDD, both the older people and their families generally tend to have poor quality of life. The growing demands for older care service, medical treatment, nursing and rehabilitation care, and an older people-friendly environment have placed an enormous burden on health services, older care services, and social security. Additionally, the COVID-19 pandemic also severely affected the health of OPWDD, increasing the burden of their caregivers. At present, the system for long-term care (LTC) policy development and implementation is fragmented and faced with challenges, such as shortage of LTC beds, insufficient beds at public institutions, and generally low occupancy rate at private institutions. There is an increasingly prominent contradiction between the ever-growing need for multi-level care services and imbalanced and inadequate service delivery. LTC is the last security in the life cycle of OPWDD, in addition to the basic older care service provided by the government as a “minimum” guarantee. Strategies to effectively solve the ever-growing needs of LTC for OPWDD have become a prominent public policy priority issue in China’s proactive response to population aging (8–14).

The Chinese government has consistently attached great importance to building the LTC system. A three-tier LTC system that covers older people was established in the 12th 5-Year Plan period (2011–2015). The Chinese central government has successively issued the *Outline of the Healthy China 2030 Plan*, the *National Medium- and Long-Term Plan for Responding Proactively to Population Aging*, and the *13th* (2016–2020) and *14th* (2021–2025) *5-Year National Plan on the cause of Aging Development and Social Older Care Service System*, as part of a national strategy for responding proactively to population aging. These initiatives integrated the concept of active and healthy aging into economic and social development, strengthening institutional innovation, policy support, and financial input. It also sought to improve the LTC system, establish an older care service system that coordinates care in homes, communities, and institutions, and integrate healthcare with LTC across care settings (*yi-yang-jie-he*) (1, 15). In 2012, cities such as Shanghai, Qingdao, and Nanjing began to explore insurance systems such as social health insurance, LTC nursing insurance, and a means-tested model (16, 17). The *Outline of the 13th 5-Year Plan* (2016–2020) *of the People’s Republic of China* issued by the State Council in 2015 explicitly called for actively exploring the establishment of an LTC insurance (LTCI) system. In June 2016, the Chinese government launched the *Guiding Opinions on Launching the Pilot Program of Long-Term Care Insurance System*, which was implemented in 15 cities, including Shanghai, Qingdao, and Ningbo (18). In September 2020, the LTCI pilot program was expanded to 49 cities across the country (19). Du et al. discussed the population background, current situation, achievements, and issues surrounding the LTC security system in China, and made certain recommendations (9, 13). Sun et al. proposed an essential care service package for PLWD during the COVID-19 pandemic. It comprised 30 service items to ensure that all PLWD enjoyed equal care services (20). Sun et al. discussed the financing models for the essential care service package for PLWD in China, estimating its affordability and sustainability. The total cost of implementing the essential care service package for PLWD in China is expected to touch 104.62 billion, 307.1 billion, and 1977.54 billion by 2020, 2030, and 2050, respectively. This would represent 1.93%, 3.32%, and 9.96% of general public budget expenditure by 2020, 2030, and 2050, respectively. These are reasonable increases in cost and ensures that the government’s public financial burden is within an acceptable range and the Chinese government has the financial capacity to ensure the sustainable operation of an LTCI system for PLWD (21). Lu et al. analyzed the Qingdao Long-term Care Medical Insurance pilot program financing, service model, and effectiveness. The program enables older people with disabilities to receive healthcare and social care at institutions or their homes, at an affordable cost, which makes it convenient and sustainable (17). Zhang et al. gathered data from 1,500 older people aged 60 and over at 15 LTCI pilot cities in China, to assess the performance of the program and measure the willingness of residents, before it is expanded across China (22). Liu et al. built a set of service capability indicators for LTC facilities using the Delphi method. The index system is composed of 6 secondary and 31 tertiary indicators. These indicators can be used to compare different LTC facilities and provide the basis for further development of capability assessment tools (23).

To the best of our knowledge, most existing studies on the construction of the LTC system in China mainly involve reports on specific aspects of the LTC, such as its institutional framework, standards, operating mechanisms, or management approaches, or provide an outcome evaluation of LTCI programs in pilot regions. There are limited reports that systematically evaluate the overall performance of the LTC system.

Identifying critical sample cases is crucial to this analysis because they are likely to be generalizable. Located on the developed eastern coast of China, Zhejiang Province recorded a per capita gross domestic product of USD 17,520 as of 2021 (24), thereby being categorized as a high-income region by the World Bank based on a gross national income per capita of USD 13,206 or more in 2022 (25). The Province has made significant progress in exploring and solving the issue of asymmetrical and inadequate economic and social development. In May 2021, the Chinese government authorized the Province to implement high-quality development and construction of a demonstrative zone for common prosperity (26). LTC for OPWDD is one of the fundamental public services provided by the government as a “minimum” living guarantee. Improving the older care service system is among the top priorities of the government to implement high-quality development and construction of a demonstrative zone for common prosperity. The key is to build a people-centered, equitable, accessible, and affordable LTC system that covers all OPWDD.

The older population of Zhejiang has grown expeditiously since 1990, about a decade ahead of the national level. The proportion of the registered population aged 60 and over in the Province exceeded 20% (20.2%) for the first time in 2015, indicating moderate aging. The registered population aged 60 and over was 12.06 million, representing 23.66% of the total population of the Province as of 2021, and a 4.17 million (7.06% of the total population) increase from 2010 (7.89 million, 16.6%). The population aged 80 and over grew from 1.21 million in 2010 to 1.81 million in 2021, representing a net growth of 6 million (49.80% of the total population). The proportion of the total registered population of the Province aged 60 and over was 4.76% higher than China’s average (18.9%). The Province ranks among the top in China in population aging, which includes a large proportion of disabled and dementia and empty-nest older population. By 2025, the total registered population aged 60 and older in the Province is expected to reach approximately 15 million, accounting for about 28% of its total population. The proportion of the OPWDD population is also expected to register rapid growth (27–29). A survey conducted by the Health Commission of the Province in 2018 revealed that 6.94% of the population aged 60 and over in the province was disabled or semi-disabled (30). The *2018–2019 Survey Report on Long-term Care in China* found that the proportion of older population with disabilities reached 11.8% (31). Accordingly, the total number of older people with disabilities in Zhejiang is estimated to range between 0.84 million and 1.42 million in 2021 and between 1.04 million and 1.77 million in 2025. Xu et al. estimated that the overall prevalence of dementia among people aged 60 and over was 5.8% (32). The total number of PLWD in Zhejiang is therefore expected to reach 6.99 million in 2021 and 8.70 million in 2025. The construction of an LTC system for OPWDD has therefore become imperative in Zhejiang’s proactive response to population aging in the new era demanding prompt solution.

In order to proactively meet the needs of LTC for OPWDD, the Provincial government of Zhejiang has been constantly strengthening policy planning and guidance and integrating resources, while striving to develop older care services in institutions, communities, and homes. Ningbo City in Zhejiang Province was one of the first 15 cities in China to implement the pilot LTCI program, and the Provincial government incorporated four neighboring cities including Jiaxing, Wenzhou, and Yiwu, as well as Tonglu County into this pilot program at the Provincial-level based on actual resource allocation, permitting extended study of LTCI coverage, fundraising, and payment of insurance benefits. Consequently, a complete and people-centered LTC system that can adapt to regional economic and social development has been gradually established in Zhejiang (27).

This study chose Zhejiang as the case study sample because the Province has set a typical example of how to build an LTC system for OPWDD in China. As Zhejiang is one of the most demonstrative Provinces for high-quality LTC development in China, its successful development of the LTC system for OPWDD proved to be replicable in other regions. The problems experienced by Zhejiang during this implementation are likely to be relevant to other cities in China. This paper analyzed the trial performance evaluation model of an LTC system for OPWDD in Zhejiang, systematically evaluated its effectiveness and overall performance from 2015 to 2021, identified weak links and influencing factors, and explored the ideal approach for developing a high-quality LTC system.

## 2. Materials and methods

### 2.1. Selection and identification of evaluation indicators

By referring to the Global Dementia Observatory framework of the World Health Organization (WHO), prior studies on LTC service evaluation (23, 33, 34) and policy papers such as the *Healthy China Initiative* (2019–2030) and *Opinions of Addressing Population Aging in the New Era*, this study constructed a performance evaluation model for China’s LTC system for OPWDD based on three dimensions: input, process and outcome, beyond the scope of traditional older care services. Evaluation indicators for the pre-selection pool were proposed. A three-level LTC performance evaluation indicator system composed of nine secondary indicators and 24 tertiary indicators was creased based on expert consultation and existing statistical statements. There are 11 tertiary indicators in input, 8 in process, and 5 in outcome. Input indicators mainly reflected the manpower, financial resources, equipment and facilities required for LTC development, process indicators that embody its fairness, accessibility, and coordination and utilization efficiency of LTC services. Meanwhile, outcome indicators described the affordability and satisfaction of LTC services, and health level.

### 2.2. Data sources

The data for this study were collected using the following resources.

#### 2.2.1. Empirical data on performance indicators of the LTC system

We employed a field social survey to collect annual panel data, including policy papers, statistical yearbooks, and work reports on LTC published from 2015 to 2021 from the Zhejiang Provincial Bureau of Statistics, Zhejiang Civil Affairs Bureau, Health Commission of Zhejiang Province, Healthcare Security Administration of Zhejiang Province, and other relevant government departments. Missing values in this paper were deducted using data from adjacent years and annual average rates.

#### 2.2.2. National policy documents on the LTC system

Policy papers from the Chinese government on LTC for OPWDD were collected from the websites of the State Council, National Health Commission, Ministry of Civil Affairs and other relevant government departments, including those describing relevant legislation, institutional norms, service standards, and the pilot operations of LTCI systems.

#### 2.2.3. International policy documents on the LTC system

International policies and regulations, such as those on legislation, institutional norms, service standards, and personnel training, were collected from the websites of the WHO, World Bank, OECD, and other relevant international organizations (9). These were then compared with related LTC policies and performance indicator values in Zhejiang Province.

### 2.3. Calculation of indicator values and trend analysis

The required indictor data were extracted and recorded by two researchers into two separate databases (Microsoft Excel 2010, Redwood, WA, USA). The consistency of the two databases was logically verified by a third researcher. Inconsistencies were addressed *via* discussions. As a reflection of the status of Zhejiang Province during the final phase of its 12th 5-Year Plan (2011–2015), 2015 data were used as baseline data for performance evaluation. The period from 2016 to 2020 represented a development stage, covered by the 13th 5-Year Plan. The year 2021 marked the commencement of the 14th 5-Year Plan (2021–2025). Basic indicators such as absolute value, constituent ratio, increase/decrease rate, and growth rate were used to conduct a descriptive analysis of trends in the performance of LTC system for OPWDD in Zhejiang from 2015 to 2021, and to evaluate corresponding outcomes.

## 3. Results

### 3.1. Changes in input indicators of long-term care for older people with disabilities and dementia in Zhejiang from 2015 to 2021

From 2015 to 2021, the Provincial government of Zhejiang consistently improved the top-level design of LTC policies and focused on development guided by planning. The construction of older care facilities had been listed for consecutive years as practical projects for improving livelihood and was included in the performance appraisal of governments at all levels. Zhejiang increased investment, strengthened total factor integration and coordination among departments, and supported various market entities in boosting effective supply in this field. Table 1 demonstrates that the proportion of LTC expenditure in the general public budget expenditure of the government of Zhejiang rose from 15.46% in 2015 to 19.92% in 2021, representing a 28.86% increase. Various policies for the introduction and training of care personnel were promulgated, such as training programs for professionals in short supply related to older caregivers, older nursing care and rehabilitation, and a hierarchical and classified LTC personnel training system. With caregivers for the older population included in provincial skills competitions, subsidy policies for employment in older care institutions were implemented, and a special post allowance system for older caregivers was established (27, 35), improving the allocation of LTC personnel. Table 1 shows that by 2021, the proportion of seniors (technician-level) among total older caregivers, and the number of social workers, and general practitioners for every 1,000 people aged 65 and over in Zhejiang was 22.47%, 12.56, and 3.77, respectively, which was 13.09%, 10.09, and 0.45 more than in 2015, yielding a growth rate of 133.96%, 408.17%, and 13.73%, respectively. Although the number of older caregivers per 1,000 people aged 65 and over increased during the 13th 5-Year Plan (2016–2020) compared with the 12th 5-Year Plan (2015), it generally declined from 7.22 in 2016 to 5.47 in 2020, and 4.78 in 2021, representing a decline of 24.25% and 33.87%, respectively (Table 1).

**TABLE 1:** Changes in input indicators of LTC for older people with disabilities and dementia in Zhejiang Province of China from 2015 to 2021.

Secondary and tertiary indicators	2015	2016	2017	2018	2019	2020	2021
**Financial security (secondary indicator)**							
Per capita disposable income of households (*yuan*)	35,537	38,529	42,046	45,840	49,899	52,397	57,541
Proportion of LTC expenditure in government’s general public budget expenditure (%)	15.46	16.83	18.40	17.86	18.00	19.53	19.92
**Human resources (secondary indicator)**							
Number of older caregivers for every 1,000 people aged 65 and over	5.08	7.22	6.91	7.06	6.67	5.47	4.78
Proportion of seniors (technician-level) in total caregivers for the older (%)	9.38	11.40	12.91	13.11	15.70	20.26	22.47
Number of social workers for every 1,000 people aged 65 and over	2.47	3.31	3.75	6.32	8.07	11.45	12.56
Number of general practitioners for every 1,000 people aged 65 and over	3.31	3.33	4.24	3.41	3.40	3.24	3.77
**Infrastructure (secondary indicator)**							
Number of older care institutions	2,240	2,365	2,183	2,207	2,382	2,299	1,860
Number of care beds at older care institutions	347,879	384,226	395,580	422,158	443,468	454,607	300,737
Number of care beds at institutions for every 1,000 people aged 65 and over	53.28	56.64	56.12	55.25	54.86	53.35	33.55
Proportion of nursing beds in total care beds at institutions (%)	40.99	43.96	46.26	46.81	53.78	51.43	54.47
Number of nursing and rehabilitation beds at medical institutions for every 1,000 people aged 65 and over	2.54	2.88	3.26	3.69	4.17	5.06	6.20

Taking advantage of the first national pilot programs for comprehensively reforming home-based and community-based older care services in China, Zhejiang strengthened coordination between institutions, communities, and homes and promoted the construction of special dementia care units in care institutions, requiring that the number of dementia care beds be no less than 5% of the total number of care beds. Zhejiang also explored the construction of older care institutions (micro institutions) embedded in communities, promoted the organic integration of formal and informal care, and offered professional support services for communities and homes (27, 36). Table 1 shows that from 2015 to 2020, the number of older care institutions, total care beds at older care institutions, and care beds at older care institutions for every 1,000 people aged 65 and over in Zhejiang increased from 2,240, 347,879, and 53.28 to 2,299, 454,607, and 53.35, respectively, representing a growth of 2.63%, 30.68%, and 0.12%, respectively. However, by 2021, the number of older care institutions (1,860), care beds at older care institutions overall (300,737), and care beds at these institutions for every 1,000 people aged 65 and over (33.55) decreased significantly by 19.10%, 33.85%, and 37.12%, respectively, compared with 2020, and 16.96%, 13.55%, and 37.05%, respectively, from 2015. The number of nursing and rehabilitation beds at medical institutions and the proportion of nursing beds at older care institutions for every 1,000 people aged 65 and older significantly grew from 2.54 and 40.99% in 2015 to 6.20 and 54.47% in 2021, respectively, representing a growth rate of 144.14% and 32.87%, respectively (Table 1).

### 3.2. Changes in process indicators of long-term care for older people with disabilities and dementia in Zhejiang from 2015 to 2021

In 2015, Zhejiang promulgated China’s first local regulation on comprehensive social services for older people, and established six standards related to rating the service criteria provided by older care institutions. High priority was accorded to the construction of institutions engaged in medical care, rehabilitation, and nursing for older people. Primary-level medical institutions were encouraged to establish rehabilitation ward for older people using idle beds, thereby constantly increasing the proportion of available rehabilitation and nursing beds and improving the three-level older health service supporting system. A linkage mechanism for the two-way referral between medical institutions and older care service institutions focusing on townships (communities) was established and improved to offer multi-level services that combined healthcare and LTC in institutions, communities, and homes. A system for older people to sign up for family doctor services was built and optimized to introduce hospital resources into communities and homes and eliminate barriers to providing older people with consistent, comprehensive, convenient, customized, and refined basic health services. The fairness and accessibility of care services were significantly enhanced over time (27, 36). Table 2 shows that by 2021, the income ratio of urban-to-rural residents (rural income as 1) in Zhejiang decreased by 6.10%, from 2.07 in 2015 to 1.94 in 2021. Furthermore, the per capita fundraising level for basic medical insurance and the participation rate of registered residents in basic medical insurance increased from 927 *yuan* and 95.00%, respectively, in 2015 to 1,379 *yuan* and 99.76% in 2021, respectively, representing increases of 48.76% and 5.01%, respectively. Older people in urban and rural areas were fully covered by basic care services. Healthcare visit rates at the national and primary levels increased by 5.06% and 3.88%, respectively, from 84.90% and 49.95% in 2015 to 89.20% and 51.89% in 2021. The rate of older people signing up for family doctor services and rate of health management for older people reached 87.30% and 70.91% in 2021, respectively, representing a 60.74% and 10.33% increase from 2015 (Table 2).

**TABLE 2:** Changes in process indicators of LTC for older people with disabilities and dementia in Zhejiang Province of China from 2015 to 2021.

Secondary and tertiary indicators	2015	2016	2017	2018	2019	2020	2021
**Service fairness (secondary indicator)**							
Ratio of urban-to-rural resident income (rural income as 1)	2.07	2.07	2.05	2.04	2.01	1.96	1.94
Per capita fundraising level of basic medical insurance (*yuan*)*	927	1,180	1,309	1,317	1,356	1,474	1,379
Participation rate of registered residents in basic medical insurance (%)	95.00	95.00	98.96	99.21	99.61	99.75	99.76
**Service accessibility (secondary indicator)**							
Visiting rate at the county level (%)	84.9	84.9	85.8	86.3	88.3	88.9	89.2
Visiting rate at the primary level (%)	49.95	50.53	50.04	51.6	52.67	53.64	51.89
Rate of family doctor services signed up for older people (%)	54.31	54.66	72.43	75.18	81.11	84.03	87.30
Rate of health management for older people (%)	64.27	65.90	68.46	68.56	69.20	70.91	70.91
**Service efficiency (secondary indicator)**							
Rate of care bed utilization at older care institutions (%)	45.87	40.93	38.50	38.97	33.27	33.20	35.90

*Data from Ningbo City, Zhejiang Province, China.

### 3.3. Changes in outcome indicators of long-term care for older people with disabilities and dementia in Zhejiang from 2015 to 2021

From 2015 to 2021, Zhejiang established and optimized a government-led diversified care supply system to provide basic care services supported by public finance for people who received subsistence allowance from the government, to set up government-supported market-oriented care services for ordinary people, and offered inclusive governmental care services for all OPWDD. As the pilot programs of the LTCI system at national and provincial levels were promoted in five cities (including Ningbo) across China, organizations that assessed the level of care needs of OPWDD basically achieved mutual recognition, unified standards, and shared results. The practice framework of the LTCI system was basically established over the course of the 5-year pilot. The number of insured people reached 8.77 million, benefiting 50,000 people overall. The insurance fund paid a total of 782 million *yuan*, covering an average of 15,600 people every year. Work, policy, indicators, and evaluation systems for LTC were consistently upgraded and the affordability and satisfaction of LTC services continued to improve (27). Table 3 shows that by 2021, the actual reimbursement ratio of hospitalization expenses covered by basic medical insurance for older people in Zhejiang increased from 72.76% in 2015 to 77.89% in 2021, representing a growth rate of 7.05%. The proportion of out-of-pocket (OOP) payment in total health expenditure (THE) decreased by 17.97%, from 29.49% in 2015 to 24.19% in 2021. Satisfaction with social security increased by 12.4%, from 71.3% in 2015 to 83.7% in 2021. Health benefits were continuously improved, and as a result, the unconditional probability of dying between 30 and 70 years of age from cardiovascular disease, cancer, diabetes, or chronic respiratory diseases decreased consistently from 11.00% in 2015 to 9.69% in 2021, an overall decrease of 4.31%. Life expectancy at birth rose from 78.22 years in 2015 to 82.20 years in 2021, representing an increase of 3.98 years.

**TABLE 3:** Changes in outcome indicators of LTC for older people with disabilities and dementia in Zhejiang Province of China from 2015 to 2021.

Secondary and tertiary indicators	2015	2016	2017	2018	2019	2020	2021
**Service affordability (secondary indicator)**							
Actual reimbursement ratio of hospitalization expenses covered by basic medical insurance (%)	72.76	72.86	73.06	74.32	77.90	77.90	77.89
Proportion of OOP payment in THE (%)	29.49	27.99	26.99	26.64	25.22	24.66	24.19
**Satisfaction (secondary indicator)**							
Social security satisfaction (%)*	71.30	71.30	73.40	75.70	82.50	83.70	83.70
**Health level (secondary indicator)**							
Unconditional probability of dying between 30 and 70 years of age from cardiovascular disease, cancer, diabetes, or chronic respiratory diseases (%)	11.00	10.68	10.18	9.67	9.04	8.99	9.69
Life expectancy at birth (years)	78.22	78.4	78.6	78.77	79.13	79.47	82.20

*Data from Ningbo City, Zhejiang Province, China.

## 4. Discussion

As suggested by international experience and relevant prior studies, a people-centered, consistent, and holistic LTC strategy is optimal for addressing China’s older care dilemma. While improving the competitiveness of care service institutions, and sharing care resources and risks, such a strategy can enhance the quality of LTC services, improve the fairness and accessibility of care services, and increase satisfaction, all of which are in line with the current goals of the global LTC system reform actively advocated by the WHO (7, 8, 33, 37, 38). According to a report jointly published by the WHO and Alzheimer’s Disease International in 2012, dementia has become a public health priority because of its high prevalence and huge economic burden on families, caregivers, and communities (39). Seven action areas and corresponding global targets for dementia care to be achieved by 2025 were proposed in *The Global Action Plan on the Public Health Response to Dementia* (2017–2025) that was adopted by the WHO in 2017. These largely focus on dementia risk reduction, patient diagnosis rates, support for caregivers and families, and training rates. The WHO called on countries to take action to develop their own national dementia support plans (40). By May 2022, only 50 WHO member-states (26% of the total) had developed their own national dementia action plans (independent plans, plans integrated into other plans, or plans below the national level), and issued related programs, policies, and strategic measures (33, 38). The LTC systems in the UK, Germany, and Japan come with personnel and mechanisms dedicated to service evaluation and care planning, thereby ensuring the integration and effective utilization of care resources. To offer social support for dementia, the UK introduced a national dementia prevention and treatment strategy that mobilizes and integrates resources for community and individual support, general practices, and family caregivers. Germany has established a community-based regional dementia care network that gives patients access to personalized services and support. The public LTCI system introduced in Japan in 2001 provides universal health insurance coverage with multiple health insurance programs, covered up to a maximum number of benefits according to the level of care required. The Japanese Ministry of Health, Labour and Welfare announced a “*Five-Year Plan for Promotion of Measures against Dementia” (Orange Plan)* in September 2012. In 2019, the government launched a new policy program for dementia care called *Comprehensive Strategy to Accelerate Dementia Measures (the New Orange Plan)* with the aim of dementia prevention and development of dementia-friendly communities (41, 42). In the USA, a people-centered LTC model has been employed for over three decades. The Program of All-Inclusive Care for the Older (PACE) has been in place since the 1970’s to address challenges with the LTC service system. PACE programs have been proven to be effective and beneficial (43–45).

Zhejiang placed high priority on population aging close on the heels of the Chinese government declaring it as a national strategy in 2020. In this paper, the current situation, achievements, and performance of the LTC system were evaluated systematically in three dimensions: input, process, and outcome, based on an empirical analysis of the construction of the LTC system for OPWDD in Zhejiang from 2015 to 2021. Such an analysis can assist with resolving key problems in the LTC system for OPWDD, build a fair and affordable LTC system with a wide coverage net, emphasize the symbolic achievements of “Longevity in Zhejiang” in the development and construction of a high-quality demonstrative zone for common prosperity, and provide an exemplary, pioneering provincial model for China to actively respond to population aging.

Our survey found that there were 53 to 56 beds for every 1,000 people aged 65 and over at older care institutions in Zhejiang from 2015 to 2020. In 2021, however, the number of older care institutions and care beds declined, driven by factors such as the renovation of fire-fighting facilities and nursing home upgrading. The number of care beds at older care institutions for every 1,000 people aged 65 and over dropped to 33.55, although it was 8.44 beds higher than the national average (25.11 beds) in 2021 (2, 3), 12.05 beds lower than the average of OECD member-states (45.6 beds) in 2019 (5). The figure is also 48.05 beds lower than that of Luxembourg, which has the highest ratio (81.6 beds), and 26.85 beds and 1.55 beds lower than those of Republic of Korea (60.4 beds) and Japan (35.1 beds), respectively. However, it is higher than that of eight countries, including the USA (32.3 beds) (5). The utilization rate of care beds at older care institutions in Zhejiang also declined by 12.67%, from 45.87% in 2015 to 35.90% in 2021, likely reflecting the low utilization rate of care beds at older care institutions during the COVID-19 pandemic. Specifically, it reveals weak links such as insufficient supply chain and supply–demand imbalance of care beds, with an imbalanced allocation of care beds in different areas due to a lack of functional integration between medical care, healthcare, nursing, rehabilitation, and other functions. The shortage of care beds in high-class public institutions in city centers was in sharp contrast with the high bed vacancy rate in remote areas and some private institutions. Excessive daily life care services also stand in stark contrast to the lack of rehabilitation and nursing services, making it difficult to effectively meet the targeted care needs of OPWDD (9, 13, 27, 36, 46). Accordingly, it is recommended that the government establish a high-quality and an integrated LTC system that focuses on maintaining *status quo*, provide support for the construction of multi-level integrated healthcare with LTC across care settings (*yi-yang-jie-he*), build additional care institutions and upgrade existing ones, promote the construction of special care units for older people with dementia patients, and increase the proportion of rehabilitation and nursing beds for older people. Moreover, standardized construction of community hospitals, dementia-friendly communities, and mobile care services should be encouraged, to introduce more embedded, multifunctional and comprehensive care facilities into communities and homes. The introduction of home-based care beds should be accelerated. A mechanism for care allocation, coordination, and referral among homes, communities, and institutions should be established and improved, so as to extend professional care services to homes, alleviate pressure related to the structural shortage of care beds, and seamlessly meet the care needs of OPWDD at different stages, thus achieving holistic, whole-life course care services for OPWDD (9, 27, 36, 47).

In more than half of OECD member-countries, population aging has been surpassing the increase of LTC supply. For example, LTC workforce has been burdened or is declining, even in countries where LTC supply is much more sufficient than the OECD average (such as Norway and Sweden) (5). This data is similar to the results of this study. The current study found that the number of older caregivers for every 1,000 people aged 65 and over in Zhejiang increased during the 13th 5-Year Plan (2016–2020) compared with the 12th 5 Year Plan (2015) period, but declined overall. By 2021, the number of older caregivers for every 1,000 people aged 65 and over was only 4.78, about 10 times lower than the average (i.e., 50) of OECD member-states in 2019 (5), suggesting a relatively large shortfall in the number of older caregivers in Zhejiang. Older caregivers were generally older themselves and less educated and professional, with few achieving senior (technician-level) rank. Such workers also lacked a sense of professional identity and service capacity, with limited potential for occupational mobility. It was consequently difficult to recruit, retain, and allocate caregivers at primary levels (10, 27, 36, 48). It is therefore suggested that a diverse range of care personnel engaged in public health, medical care, geriatric care, rehabilitation and nursing, and hospice care be allocated in a coordinated manner. The engagement of charity organizations, social workers, and volunteers in LTC services should be comprehensively promoted. Training programs for top-notch personnel in LTC and upcoming technical personnel should be implemented to strengthen the knowledge and skills of on-the-job personnel in disability and dementia care service. A policy system to incentivize care personnel should be established, such as offering “rewards and subsidies upon employment,” continuously improving the remuneration, development space, and social status of care personnel (5, 9, 27, 35, 36, 46, 47).

The proportion of OOP payment in THE is a primary indicator of a household’s medical burden and reflects their affordability of public health. Households with a member who has disabilities or dementia are more likely to face increasing health expenditures. In *The World Health Report 2010. Health Systems Financing: the Path to Universal Coverage*, the WHO points out that it is only when the confidence on OOP payment as a proportion of THE declines to less than 15–20% that the incidence of financial burden or impoverishment routinely falls to negligible levels. Long-term goals should focus on reducing the proportion of OOP payment in THE to below 15–20% (49). Zhejiang has enhanced its protection of the population from the risk of high OOP healthcare costs in recent years. The share of OOP payment in THE has significantly reduced year-on-year, from 29.49% in 2015 to 24.19% in 2021. These figures are both below the target set in *The Healthy Zhejiang Action Plan* (2030), which established that the “proportion of OOP payment in THE should be reduced to about 28% by 2020 and about 25% by 2030 (50),” which is 3.41% lower than the Chinese average (i.e., 27.60%) in 2021 (2). It is nevertheless above the WHO’s recommended level of 20% for reducing the risk of impoverishment as a result of disease (49). It was also higher than the average of all OECD member-states (20%), Australia (18%), Germany (13%), and Japan (13%) in 2019 (5). This suggests that there is still a significant risk of catastrophic health expenditure for households with OPWDD in Zhejiang. Additionally, OOP payment burden needs to be further alleviated through comprehensive measures for healthcare and LTC. Such medical burdens should be alleviated by “precise identification and targeted assistance.” A multi-level medical insurance system should be established and improved. As indicated from the experience of the five cities that participated in the national and Provincial pilot program of the LTCI system, a similar system involving the government, employers, and employees should be gradually established and promoted Province-wide. The construction of a diversified health financing mechanism that is mainly funded by governmental subsidies and shared by enterprises, charitable donations, and individuals should be constantly expanded. Efforts should also be made to provide further flexibility in the pilot programs of the LCTI system. TCLI financing and coverage should be constantly expanded to cover all insured people. All people entitled to the insurance should be covered. The share of OOP payment in THE should be continuously reduced so as to protect against health payment risks and prevent households from catastrophic health expenditure (49, 51).

### 4.1. Strengths and limitations

Previous studies mainly focused on the Chinese LTC system from the theoretical perspective of LTC service supply, care needs, and LTCI pilot program assessment from a single aspect (9). To the best of our knowledge, this paper may be the first to evaluate the input, process, and outcome of the LTC system in China in order to assess its performance, issues, and countermeasures. Our study has two highlights. First, it takes on one of the largest-scale efforts to construct a set of performance evaluation indicators suitable for the LTC system among OPWDD in China. A well-validated data register system is used from the Bureau of Statistics, Health Committee, Civil Affairs Bureau, and Medical Security Bureau of Zhejiang Province, China, on behalf of the most authoritative sources from which performance evaluation indicator data could be collected. Second, the study provides a significant body of information to permit a comprehensive understanding of the current performance of the LTC system, which may help policymakers, practitioners, and researchers to identify its strengths, weaknesses, and areas for improvement.

Several potential limitations should be considered. First, although the performance evaluation indicators for OPWDD were selected based on a comprehensive literature review in addition to specialist points of view and realistic conditions, the evaluation model was tested in Zhejiang Province using empirical data from 2015 to 2021 that basically conformed to the reality. The LTC system is influenced by many factors, such as politics, economy, population, culture, health and social security system. The inconsistency of the cognitive dimensions and social value orientation for the LTC system between different regions can lead to misunderstanding. Some individual indicators, such as life expectancy and healthy life expectancy at age 65, the early diagnosis rate of dementia, number of care beds in special care units for dementia and their utilization rate, and the involvement of people with disabilities and dementia in research and innovation, could not be used due to a lack of relevant available empirical data (34). These evaluation indicators should be further enhanced in future studies. Second, the analysis is based on data from only Zhejiang Province. While Zhejiang’s experience may be representative of LTC programs in other cities that were similarly designed, caution should be exercised while generalizing the study results to China overall as there are considerable differences in regional economic development and social-demographics. Data from a single wealthier developed sample province on eastern coast China may limit the generalization of the results. Finally, this study is predominantly cross-sectional and lacks controlled policy intervention trials, which may reduce its validity.

Despite the limitations, this study presents crucial knowledge and insight to policymakers and stakeholders. With more empirical data likely to be available in the future, we will carry out further quantitative and qualitative comparative research using classification and grading indicators on an expanded sample size, possibly permitting the execution of a controlled evaluation of a person-centered, integrated LTC system. Such work would help us formulate a scientific, practicable, and effective quantitative management means for measuring the performance of an LTC system among OPWDD, which deserves application on a large scale (20, 31).

## 5. Conclusion

In summary, the coverage and utilization of the LTC system in China has consistently increased. This article presents a novel perspective to help us understand the initial value and efficacy of an LTC system focusing on input, process, and outcome in Zhejiang Province, China. It used an innovative computational approach method as a policy evaluation tool to offer evidence-based recommendations on the LTC system to policymakers in China. This study makes two contributions: First, we have built a set of performance evaluation indicators suitable for the LTC system for OPWDD in China. Second, this paper discusses the background of aging of the Chinese population and the current performance situation and achievement of the LTC system in Zhejiang. The results of this study are expected to provide an empirical basis data for policymakers, practitioners, clinicians, and researchers to establish an effective performance assessment tool of the LTC system focused on OPWDD, which may contribute to policy optimization. Essentially, the insights gleaned from this case study could increase awareness of successful LTC policy implementation in developed regions of China, supporting a nationwide action plan for developing a feasible, diversified integrated health and social care system for OPWDD in the future for policymakers in both China and low- and middle-income countries, especially those with a large older population.

## Data availability statement

The original contributions presented in this study are included in the article/supplementary material, further inquiries can be directed to the corresponding author.

## Ethics statement

The study protocol was approved by the Ethics Committee of Ningbo College of Health Sciences, China (approval number LG2023008).

## Author contributions

TS, XL, and LL: conceptualization, writing—review and editing, and resources. TS, WJ, XD, and MJ: data curation and investigation. TS and XD: formal analysis. XL and LL: funding acquisition, project administration, and supervision. TS and LL: methodology and validation. WJ, XD, and MJ: visualization. TS, XL, WJ, XD, and LL: writing—original draft. All authors read and agreed to the published version of the manuscript.
